# Health Care Transformations Merging Traditional and Digital Medical Practices

**DOI:** 10.1016/j.mcpdig.2023.02.006

**Published:** 2023-03-25

**Authors:** Jon O. Ebbert, Rita G. Khan, Bradley C. Leibovich

**Affiliations:** a Community Internal Medicine, Geriatrics, and Palliative Care, Department of Medicine, Mayo Clinic, Rochester, MN; b Center for Digital Health, Mayo Clinic, Rochester, MN; c Department of Urology, Mayo Clinic, Rochester, MN

Digital transformation enables the delivery of efficient, high-quality, and secure health care,^1^ holding tremendous promise for the development and deployment of new care models. Digital transformation can evolve and expand clinical care processes through information, computing, communication, and connectivity technologies.^2^ Digital transformation leveraging emerging technologies can enhance health care organizational efficiencies and transform patient care models through patient empowerment.^3^

Digital transformation in business has affected consumer expectations, disrupted major markets, and placed significant pressure on traditional companies.^4^ Traditional medicine is facing potential disruption with the entry of digital health care companies; however, significant opportunities exist to extend and improve patient care through innovative partnerships between traditional and digital providers. In this commentary, we present potential models for these collaborations and current challenges and approaches to overcome them.

### Current Health Care Landscape

Primary care is day-to-day health care delivered to patients by medical providers. Secondary care encompasses services delivered by medical or surgical specialists. Tertiary care is provided by hospitals or surgical centers. Health care value-chain activities provided in these settings include monitoring/preventing, diagnosing, preparing, intervening, recovering/rehabilitating, and monitoring/managing.^5^ Health care providers are practicing in an environment with increased demand for these value-chain activities, an aging population, and downward pressures on reimbursement. All providers share responsibility for these value-chain activities, which are commonly poorly coordinated across multiple, unaffiliated health care institutions.

Primary care providers (PCPs) are currently challenged to meet patient needs. A simulation study estimated that PCPs require 26.7 h/d to provide preventive care, chronic disease management, and acute care; complete documentation; and manage data for a standard patient panel size.^6^ Specialists receiving referrals for the diagnosis and management of medically complex patients similarly experience significant strain on existing limited resources. With increasing clinical demands, rising clinician burnout, and workforce scarcity, traditional medical practices can capitalize on digital transformation to meet extant challenges while continuing to provide affordable, high-quality health care to the entire spectrum of patients.

Digital health care companies with emerging technologies have entered the marketplace with the promise of supplying on-demand services at a lower cost. Service offerings include longitudinal care, urgent care, and specialty care. Many entrants are capitalizing on artificial intelligence, telemedicine, and blockchain electronic health records (EHRs) to set up streamlined workstreams that reduce error and improve patient outcomes. Significant footholds have been established through the provision of on-demand health care coinciding with advancements in consumer smartphones. Many of these companies have not established reimbursement models with traditional health insurance companies and instead rely on fee-for-service or subscription models.

### Collaborative Models

#### Primary Care

Primary care is struggling with increasing patient medical complexity, the exodus of retiring clinicians with insufficient replacement trainees, declining reimbursement, and a data deluge accelerated by increased use of diagnostic and screening modalities. This constellation of challenges is restricting access to primary care. In this setting, technologically savvy patients are availing themselves of digital health care delivered conveniently in their work or home settings.

Traditional primary care practices can view this inexorable reality as a threat or an opportunity. Envisioning collaborative models between traditional and digital health care providers could be actualized in the domains of the clinical care value-chain activities discussed previously. We present diabetes, a common clinical condition, as a useful platform for idea exploration and propose a model for these interactions (Figure).

**Figure:**
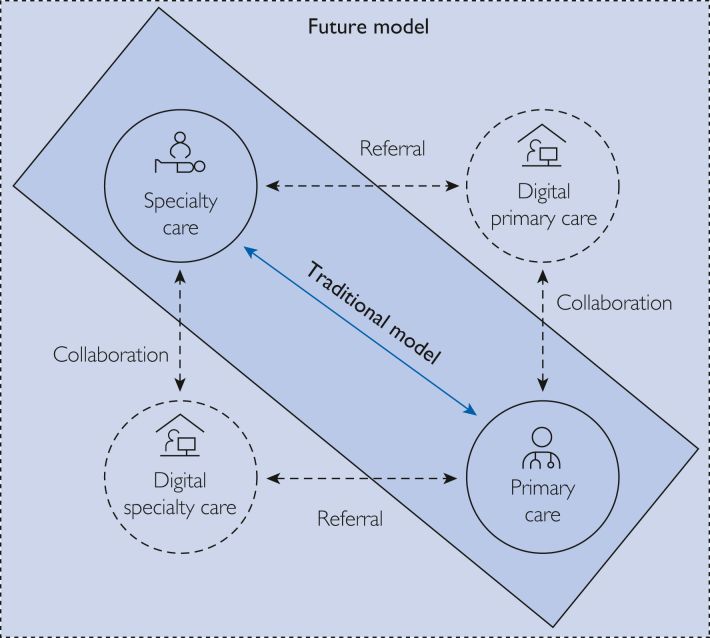
Current and future connectivity between traditional and digital care.

##### Use Case: Diabetes

The prevalence of diabetes is increasing, and diabetic care in the United States involves the evolution of more complex tasks for less reimbursement per task. Diabetes is a quality metric against which health care institutions are measured often tied to reimbursement.

###### Monitoring**/**Preventing

Both traditional and digital PCPs who have “empaneled patients” collect data that could be used to facilitate an early diagnosis of diabetes, such as age, body mass index, physical activity, race, and family history. Partnerships could promote collaboration through digital companies referring to traditional practices for laboratory testing when it is geographically proximal and more convenient for the patient than coordinating home testing through a third party.

###### Diagnosing

Once a diagnosis of diabetes is made, collaboration between traditional and digital PCPs could be facilitated by complementing each other’s resources. For example, a digital primary care company could refer to the traditional PCP for a baseline clinical diabetic foot examination or receive a referral for education in the patient home when new insulin starts after the medication is obtained by the patient. Digital primary care companies who are exclusively responsible for the patient might refer to traditional endocrinology specialty care to set up a care plan that the digital health care provider could follow.

###### Intervening

Patients with diabetes have greater health care use than patients without diabetes and frequently need clinical evaluation for related or unrelated problems. Partnerships could be built, allowing for patient triaging to face-to-face or digital experiences on the basis of patient acuity and need.

###### Monitoring**/**Managing

Both traditional and digital PCPs have the capabilities to monitor and manage diabetes. Digital companies using application-enabled or internet-of-things glucometers and wearables may provide advantages through continuous feedback, counseling, activity and medication compliance tracking, or provider recommendations for patients with poor glucose control. Traditional primary care institutions can supply added diagnostics, such as laboratory and radiology, to diagnose other causes of hyperglycemia, such as infection. Digital health care providers could refer for routine screening to traditional subspecialties (ie, ophthalmology for retinal examinations) or primary care partners (ie, diabetic foot examinations). Both types of providers may be incentivized to collaborate through comanagement of patients in an accountable care organization framework.

#### Specialty Care

Opportunities exist for specialty care health care providers to collaborate with digital health care providers both in collaborative and referral relationships (Figure). For this use case, we present inflammatory bowel disease (IBD), a complex condition often requiring advanced diagnostics and therapeutics, which are currently offered by digital health care providers in the health care marketplace.

##### Use Case: IBD

###### Diagnosing

Both traditional and digital specialty health care providers can develop high pretest probabilities for IBD on the basis of patient history alone. The diagnosis of IBD is routinely proven through clinical features, laboratory abnormalities, findings on imaging and endoscopy, and histopathologic analysis. Digital health care providers can refer to local laboratories for blood testing if convenient for patients, radiologists for imaging, and gastroenterologists for endoscopic diagnostic services, as needed.

###### Preparing

Preprocedural evaluations could be performed by digital health care providers to reduce time and expense for patients needing diagnostic procedures, such as endoscopy, performed by specialists.

###### Intervening

Both traditional and digital specialty health care providers can provide treatment for IBD. Specialty care providers have built infrastructure to supply advanced diagnostics and procedural expertise. Collaborations between both groups could center around complementing each other’s resources, such as digital specialists supplying dietary advice or medication management after treatment starts or referring to traditional specialists for enrollment in new therapeutic clinical trials.

###### Monitoring/Managing

Digital specialists may offer advantages by supplying around-the-clock access to application-based supportive care and reducing geographic barriers for patients while receiving referral “hand offs” for ongoing patient support after a diagnosis is made and a treatment course is created. Traditional specialists can receive referrals for routine surveillance colonoscopies, radiology, and laboratory diagnostics. Traditional specialists have begun and will continue to extend their previsit and postvisit presence through enhanced digital offerings, further enhancing virtual clinical engagement with digital specialty providers. Digital specialists may discover unrelated problems that they could refer for evaluation to traditional PCPs for face-to-face evaluation and treatment in the interest of patient safety.

###### Challenges and Opportunities

In this article, we present opportunities for collaborative relationships between traditional and digital medical practices and propose an emerging model. Significant challenges exist to the development of these types of partnerships. First, incentives are not aligned for the building of these partnerships in the current marketplace. Innovative revenue models are needed to allow for equitable revenue sharing. Second, concerns have been raised about digital care services increasing health care use by “lowering the bar” for seeking medical expertise. In an analysis of commercial claims for direct-to-consumer telehealth, 12% of visits replaced face-to-face visits, whereas 88% represented new utilization.^7^ This could potentially increase downstream demand for clinical services and intensify, rather than alleviate, resource constraints on traditional medical institutions. To address these issues, the advancement of technologies leveraging artificial intelligence for symptom-checking may reduce more resource-intensive visits requiring provider input, and patients have reported this type of tool to be useful.^8^ Third, the lack of universal EHR connectivity, although not a new challenge to modern medicine, could increase patient data fragmentation because each digital and traditional health care provider will “own” a separate piece of the clinical narrative. Adoption of inbound and outbound referral portals allowing access to EHRs and the use of application programming interfaces could mitigate this threat. Finally, models are needed around the optimal provision of digital technologies to older patients who may prefer receiving them supported by family members, thus making their engagement more successful.^9^

Digital transformation holds enormous potential for improving patient care at a time of increasing clinical demands, constrained resources, and declining reimbursement. Traditional and digital practices hold the key to whether they are competitors or collaborators in the future of health care. Innovative partnerships will advance the development of provider ecosystems, enabling the delivery of efficient and effective patient care.

## Potential Competing Interests

Dr Ebbert serves on a scientific advisory board for Applied Aerosol Technologies and serves as a consultant to Exact Sciences and K Health, with reimbursement paid to Mayo Clinic. Author Khan reports stocks in Servicenow, 3M, Confluent, United Airlines, Exact Sciences, and British Petroleum. Dr Leibovich, an editorial board member, had no role in the editorial review of or decision to publish this article.
